# Loss of TRPV4 is insufficient to promote repair in a spinal cord injury contusion model

**DOI:** 10.1038/s41598-025-12372-1

**Published:** 2025-07-23

**Authors:** Melanie Mertens, Sofie Kessels, Naomi Veeningen, Elle E. M. Scheijen, Femke Mussen, Amber Delbroek, Jana Van Broeckhoven, Yeranddy A. Alpizar, Bert Brône

**Affiliations:** 1 Department of Neuroscience, Biomedical research institute, Hasselt University, 3500 Hasselt, Belgium; 2 Department of Immunology and Infection, Biomedical research institute, Hasselt University, 3500 Hasselt, Belgium; 3 European Research Institute for the Biology of Ageing (ERIBA), University Medical Center Groningen (UMCG), University of Groningen (RUG), 9613 Groningen, The Netherlands; 4 Department of Psychiatry and Neuropsychology, School for Mental Health and Neuroscience, Maastricht University, 6229ER Maastricht, The Netherlands; 5 Mechanobiology & Biomaterials group, University of Mons, 7000 Mons, Belgium; 6 Laboratory of Molecular Vaccinology & Vaccine Discovery (MVVD), Department of Microbiology, Immunology & Transplantation, Rega Institute, 3000 Leuven, Belgium

**Keywords:** Spinal cord injury, Phagocytes, Transient receptor potential vanilloid 4, Contusion model, Bone marrow transplantation, Mechanisms of disease, Ion channels in the nervous system, Spinal cord injury, Cellular neuroscience

## Abstract

Microglia, the primary phagocytes activated after spinal cord injury (SCI), play a key role in containing the lesion and protecting the glial scar from infiltrating immune cells. Although these responses are initially protective, excessive microglial proliferation and sustained pro-inflammatory activation can worsen secondary damage and limit recovery. Modulating microglial activity has been proposed as a potential therapeutic approach to enhance SCI repair. Previous studies suggest that constitutive loss of the mechanosensory channel TRPV4 (transient receptor potential vanilloid 4) reduces microgliosis and inflammation at the lesion site, improving functional outcomes. TRPV4 is a Ca^2+^-permeable channel implicated in several microglial characteristics, like morphology, motility, proliferation, and phagocytosis. Whereas endogenous TRPV4-activating stimuli are abundant at the lesion site, the microglia-specific contribution of TRPV4 in SCI recovery remains unknown. To investigate this, we used phagocyte-specific *Trpv4* conditional knockout mice and phagocyte-specific TRPV4-deficient bone marrow chimeras subjected to contusion SCI. We achieved robust and efficient spinal cord monocyte-derived cell repopulation after bone marrow transplantation and PLX treatment. Surprisingly, TRPV4 deficiency in phagocytes did not enhance functional recovery, reduce microgliosis, or diminish scar formation after SCI. Furthermore, contrary to prior studies, constitutive TRPV4 deficiency did not improve SCI outcomes, indicating that the role of TRPV4 in this context is complex and potentially redundant with other pathways.

**Supplementary Information:**

The online version contains supplementary material available at 10.1038/s41598-025-12372-1.

## Introduction

Microglia, the resident immune cells of the central nervous system (CNS), are key players and the first cells to respond after spinal cord injury (SCI)^1–6^. They become rapidly activated by damage signals and interact with the injured environment via various pathways^1^. Microglia play a neuroprotective role by promoting tissue repair via the secretion of trophic and anti-inflammatory factors. They clear the environment of apoptotic cells and cellular debris via phagocytosis^3,5,7^. Additionally, these cells contribute significantly to glial scar formation by promoting astrocyte proliferation and positioning themselves along the inner border of the scar, serving as a barrier against infiltrating immune cells^5^. In contrast to these protective tasks, continuous and excessive microglial proliferation and their pro-inflammatory character exacerbate injury outcome due to the induction of chronic inflammatory cascades^2,3,6,7^. Therefore, the effect of microglia on SCI repair can be either protective or detrimental, depending on the fine balance of their functions^6,8–10^.

Upon activation, microglia extend their dynamic branches towards the insult in response to chemoattractant stimuli, such as ATP^4^. In both homeostatic and reactive states, microglia strongly depend on Ca^2+^ signaling, which is more prominent in the extending branches than their soma^11,12^. Not only chemoattractants but also mechanical stretch via mechanosensitive Ca^2+^ channels drive cellular activity^12^. Transient receptor potential (TRP) channels, a family of non-selective cation channels, are involved in cellular Ca^2+^ signaling and play a role in microglial functioning, among which TRPV4, a thermo-, osmolarity-, and mechanosensor^13–16^. TRPV4 was shown to regulate microglial migration, motility, morphology, and cytoskeletal dynamics^17–21^.

After SCI, TRPV4 expression is increased in microglial cells, among others^22^. The initial insult to the spinal cord causes a cascade of events such as cell death, cell swelling, ATP release, changes in osmolarity, shear stress, changes in mechanical properties of the tissue, and a local temperature increase due to inflammation^22–29, ^all of which act as endogenous stimuli for TRPV4^15,16,23^. TRPV4 in microglia was identified at the center of the neuroimmune axis in the spinal cord, as blocking TRPV4 channel activity attenuated neuropathic pain^30,31^. In addition, a constitutive deficiency of TRPV4 was found to be protective after compression-induced SCI and was associated with less glial and fibrotic scarring, reduced inflammation, diminished microglial activity, and improved neuronal protection and functional recovery^22^. These data suggest that increased TRPV4 channel activity after SCI evokes an excessive microglial response, leading to sub-optimal recovery. However, the role of TRPV4 in specific cell types present at the lesion, including microglia, is unknown. In this work, we refer to microglia and infiltrated macrophages as phagocytes. In our models, both immune cell types are largely indiscernible due to the expression of similar markers like CX3CR1, CD11b, IBA-1, and Tmem119^7,32–34^.

We hypothesize that a selective deficiency of TRPV4 in microglia is sufficient to improve functional recovery after SCI. To address this, we applied a contusion-induced SCI in two mouse models with TRPV4-deficient microglia. In contrast to our hypothesis, the selective deletion of *Trpv4* in the immune compartment is insufficient to enhance SCI repair. Moreover, our results suggest that a constitutive knockout (KO) of *Trpv4* does not promote SCI recovery.

## Results

### Phagocyte-specific *Trpv4* conditional knockout does not improve functional recovery after contusion SCI

**Fig. 1:**
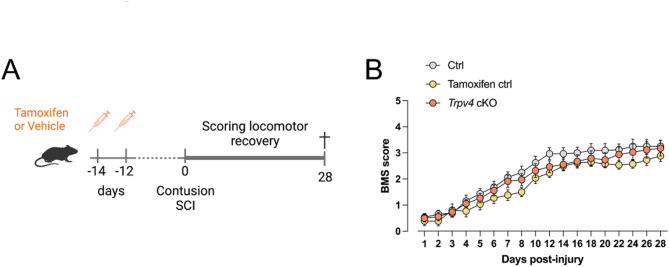
Conditional knockout of *Trpv4* in phagocytes does not improve functional recovery after spinal cord injury. (**A**). Timeline of the experimental procedures with three experimental groups (10 weeks old): (1) *Trpv4*^*lox/lox*^ *Cx3cr1*^*CreER/+*^ animals receiving corn oil injections (Ctrl); (2) *Trpv4*^*lox/lox*^ without *Cx3cr1-CreER* receiving tamoxifen injections (Tamoxifen ctrl); (3) *Trpv4*^*lox/lox*^ *Cx3cr1*^*CreER/+*^ animals receiving tamoxifen injections (57 mg/kg body weight) (*Trpv4* cKO). (**B**) Functional recovery over 28 days after contusion injury at T8 of the Ctrl (*n* = 16), Tamoxifen Ctrl (*n* = 13), and *Trpv4* cKO (*n* = 17) groups of three individual experiments. Two-way ANOVA. Data are presented as means ± SEM. Ctrl, control; cKO, conditional knockout; T8, thoracic level 8; SCI, spinal cord injury; BMS, Basso Mouse Scale.

Since Kumar et al.^22^ described the protective role of constitutive TRPV4 deficiency in SCI recovery, we were interested in the contribution of TRPV4 in phagocytes. We investigated whether a selective deficiency of phagocytic TRPV4 can improve functional recovery after SCI^22^. First, a tamoxifen-inducible myeloid-specific *Trpv4* conditional KO (cKO) mouse model was generated (Fig. 1A). TRPV4 is expressed in several CNS and peripheral macrophage populations^35^. Therefore, we validated the cKO model in both primary microglia and peritoneal macrophages and confirmed the genetic elimination (Fig. S1). Two weeks after injection, 12-week-old mice received a contusion SCI. A gradual improvement was seen over time, as all experimental groups improved from a Basso Mouse Scale (BMS) score between 0 and 1 (no/slight ankle movement) at 1-day post-injury (dpi) to a score of 3 (plantar placing of the paw with or without weight support) at 28 dpi (Fig. 1B). However, phagocyte-specific *Trpv4* cKO mice exhibit no significant differences in locomotor recovery compared to control littermates, suggesting that TRPV4 in phagocytes does not influence contusion-induced SCI recovery.

### Spinal cord-grafted TRPV4-deficient phagocytes do not improve contusion SCI outcome

**Fig. 2:**
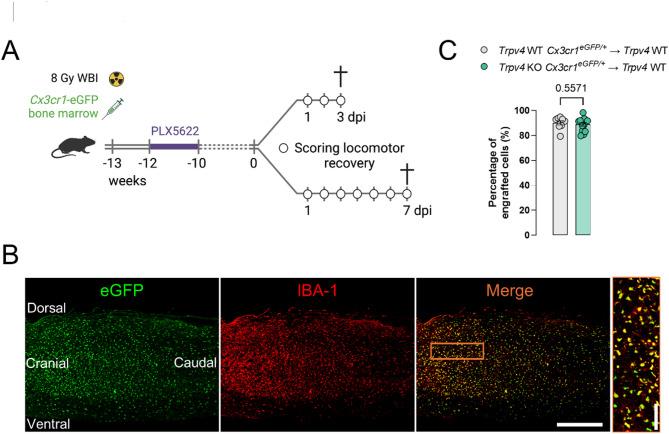
Chimerism of microglia-like cells in the spinal cord after bone marrow transplantation and PLX5622 treatment. (**A**) Timeline of the experimental procedures. 10-week-old *Trpv4* WT and *Trpv4* KO mice receive bone marrow of *Trpv4* WT *Cx3cr1*^*eGFP/+*^ or *Trpv4* KO *Cx3cr1*^*eGFP/+*^ animals and PLX5622 treatment for microglia depletion in the brain and spinal cord (1.2 g/kg chow). (**B**) Overview of eGFP^+^-repopulating donor phagocytes (eGFP), IBA-1^+^ donor and recipient phagocytes (IBA-1), and an overlay (merge) in a sagittal spinal cord 13 weeks post-BMT (no spinal cord injury). (**C**) Quantification of the repopulation of *Trpv4* WT (*n* = 9 mice) and *Trpv4* KO (*n* = 14 mice) phagocytes in the spinal cord 13 weeks post-BMT (double-positive cells). Mann-Whitney *U *test. Data are presented as means ± SEM. Scale bars: 500 μm. BMT, bone marrow transplantation; WT, wild-type; KO, knockout; eGFP, enhanced green fluorescent protein; WBI, whole body irradiation; dpi, days post-injury.

**Fig. 3:**
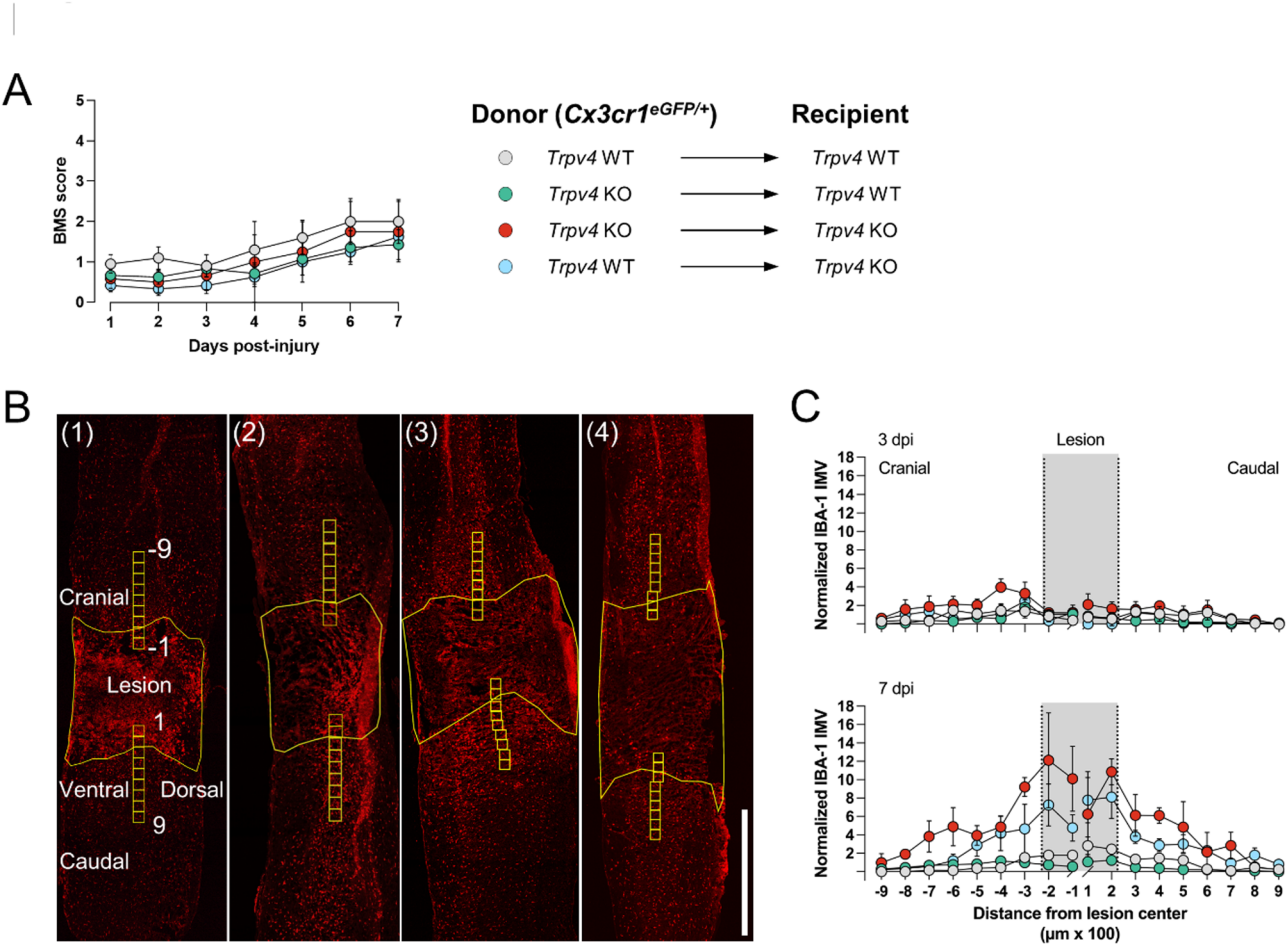
Recovery after spinal cord injury does not improve in a phagocyte-specific bone marrow transplantation model. (**A**) Functional recovery over 7 days after contusion injury at T8 of the four different BMT groups: (1) *n* = 5, (2) *n* = 7, (3) *n* = 2, and (4) *n* = 4, of 3 individual experiments. Overview of bone marrow chimera groups: (1) full WT (*Trpv4* WT *Cx3cr1*^*eGFP/+*^ → *Trpv4* WT); (2) phagocyte-specific *Trpv4* KO (*Trpv4* KO *Cx3cr1*^*eGFP/+*^ → *Trpv4* WT); (3) full *Trpv4* KO (*Trpv4* KO *Cx3cr1*^*eGFP/+*^ *→ Trpv4* KO); (4) TRPV4-expressing phagocytes in a TRPV4-deficient host (*Trpv4* WT *Cx3cr1*^*eGFP/+*^ → *Trpv4* KO). (**B**) IBA-1^+^ phagocytes in sagittal spinal cord sections at 7 dpi of all BMT groups, with an overview of the lesion and marked regions at different distances from the lesion center. (**C**) Quantification of the phagocytic density at different distances from the lesion center at 3 and 7 dpi. Two-way ANOVA (**A**) and Multiple Mann-Whitney *U* test (**C**). Data are presented as means ± SEM. Scale bar: 500 μm. T8, thoracic level 8; BMT, bone marrow transplantation; WT, wild-type; KO, knockout; eGFP, enhanced green fluorescent protein; BMS, Basso Mouse Scale; dpi, days post-injury; IMV, intensity mean value.

As we did not observe an improvement in functional recovery in the phagocyte-specific *Trpv4* cKO at 28 dpi (Fig. 1B), we aimed to validate these results using a second phagocyte-specific *Trpv4* KO model. Therefore, four groups of bone marrow chimeras were created, using a combination of bone marrow transplantation (BMT) and PLX5622 treatment, to distinguish the role of TRPV4 in phagocytic (myeloid) cells and non-myeloid cells (e.g., neurons, astrocytes, or other tissue-resident cells) (Fig. 2A). A two-week regimen of PLX5622 depletes microglia in the CNS (brain and spinal cord^5^) and allows the repopulation of bone marrow-derived cells into the brain and spinal cord^36,37^. In the first group (*Trpv4* wild-type (WT) donor → *Trpv4* WT recipient), both donor and recipient are *Trpv4* WT mice (Fig. 3A). In the second group (*Trpv4* KO donor → *Trpv4* WT recipient), the transplanted bone marrow (donor) is from *Trpv4* KO mice, while the recipient is a *Trpv4* WT mouse. This setup evaluates the specific contribution of TRPV4-deficient phagocytes to pathophysiology, leaving all other non-bone marrow-derived cells intact with functional TRPV4 channels. In group 3 (*Trpv4* KO donor → *Trpv4* KO recipient), both donor and recipient mice are *Trpv4* KO. Groups 1 and 3 are control groups that were included to assess possible BMT-induced effects and serve as a model for *Trpv4* WT and *Trpv4* KO mice. In group 4 (*Trpv4* WT donor → *Trpv4* KO recipient), the transplanted bone marrow is from *Trpv4* WT mice, while the recipient is a *Trpv4* KO mouse. This setup assesses whether TRPV4 deficiency in resident CNS cells (e.g., neurons, astrocytes, and PLX5622-resistant microglia) affects recovery, while bone marrow–derived myeloid cells retain functional TRPV4 channels (Fig. 3A)^22^. All donor mice exhibited hemiallelic expression of enhanced green fluorescent protein (eGFP) under the promoter of C-X3-C motif chemokine receptor 1 (Cx3cr1), being heterozygous for the eGFP gene. Consequently, these mice were also hemiallelic for *Cx3cr1*. This facilitates the identification of transplanted myeloid cells (GFP^+^) from the host myeloid cells (GFP^−^).

Successful repopulation was achieved in the spinal cord, proven by > 90% of microglia-like cells arising from the transplanted bone marrow at 13 weeks post-BMT (Fig. 2B, C). This was based on double eGFP/IBA-1 expression of bone marrow-derived phagocytic cells. The engrafted phagocytes were also homogeneously distributed (Fig. 2B), suggesting that the impact of TRPV4 deficiency in phagocytes is dispensable for their repopulation after BMT^21^. With an observed survival rate of 78% despite the high invasiveness, bone marrow chimeras seemed a suitable model to study recovery after SCI.

Spinal contusion surgery was performed after optimal repopulation of transplanted bone marrow cells in the CNS, i.e., 13 weeks post-BMT at 23 weeks. Locomotor recovery was assessed until day 3 and day 7 after surgery (Fig. 2A). The microglia-specific TRPV4 effect was compared at earlier time points after injury compared to the cKO model, as microglia are the first responders after SCI. All groups recovered similarly, gaining locomotion up to a BMS score between 1 and 2 (slight/extensive ankle movement) on day 7, regardless of the selective deficiency of TRPV4 in phagocytes or stromal cells (Fig. 3A). At the histological level (Fig. 3B), no difference was observed in phagocyte density around the lesion at acute time points after injury (3 and 7 days), with the highest microgliosis observed at 7 dpi, as expected (Fig. 3C)^5^.

### The absence of TRPV4 does not alter functional recovery and scar formation after SCI

**Fig. 4:**
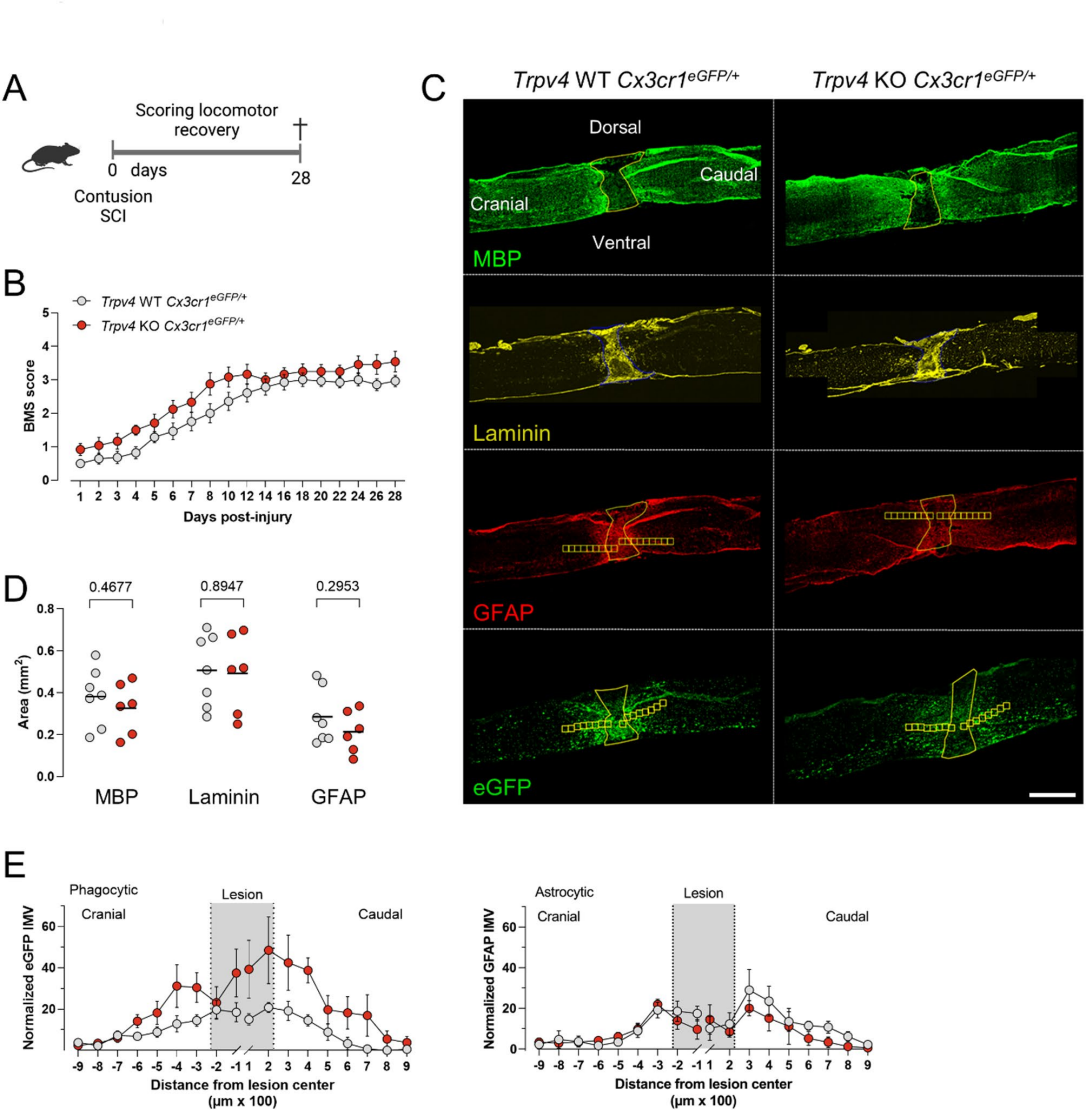
Full absence of TRPV4 does not contribute to recovery after spinal cord injury in a heterozygous model of Cx3cr1. (**A**) Timeline of the experimental procedures. (**B**) Functional recovery of 12-week-old *Trpv4* WT *Cx3cr1*^*eGFP/+*^ (*n* = 14) and *Trpv4* KO *Cx3cr1*^*eGFP/+*^ (*n* = 12) mice over 28 days after contusion injury at T8, of two individual experiments. (**C**) Overview of MBP, Laminin, GFAP^+^ astrocytes, and eGFP^+^ phagocytes around the lesion of a sagittal spinal cord section. (**D**) Quantification of lesion area surrounded by MBP, laminin, and GFAP^+^ astrocytes at 28 dpi. (**E**) Phagocytic (eGFP) and astrocytic (GFAP) density at different distances from the lesion at 28 dpi. Two-way ANOVA (**B**), Unpaired *T*-test (**D**), and Multiple unpaired *T*-test (**E**). Data are presented as means ± SEM. Scale bar: 1 mm. WT; wild-type, KO; knockout, T8, thoracic level 8; eGFP, enhanced green fluorescent protein; MBP, myelin basic protein; GFAP, glial fibrillary acidic protein; SCI, spinal cord injury; BMS, Basso Mouse Scale; dpi, days post-injury; IMV, intensity mean value.

No improvement in functional recovery nor a decrease in microgliosis was seen after SCI in the two phagocyte-specific TRPV4-deficient models. To rule out the effect of the BMT procedure on the repopulated myeloid cell characteristics, we investigated recovery after contusion SCI in full *Trpv4* KO *Cx3cr1*^*eGFP/+*^ mice of 12 weeks old (Fig. 4A). To investigate recovery at an intermediate time point in between the acute and chronic phase after SCI (i.e., 14 days to 6 months), animal follow-up was performed until 28 dpi^38^. Locomotor recovery was assessed, and both *Trpv4* WT *Cx3cr1*^*eGFP/+*^ and *Trpv4* KO *Cx3cr1*^*eGFP/+*^ mice showed gradual improvement of hindlimb movement with a BMS score of 3 (plantar placing of the paw with or without weight support) at 28 dpi without differences in recovery (Fig. 4B). At the histological level, no difference was observed in demyelination (myelin basic protein; MBP), fibrotic scar (laminin) and glial scar (glial fibrillary acidic protein; GFAP) formation (Fig. 4C, D). In addition, phagocyte and astrocytic density did not differ between the two groups (Fig. 4E).

**Fig. 5:**
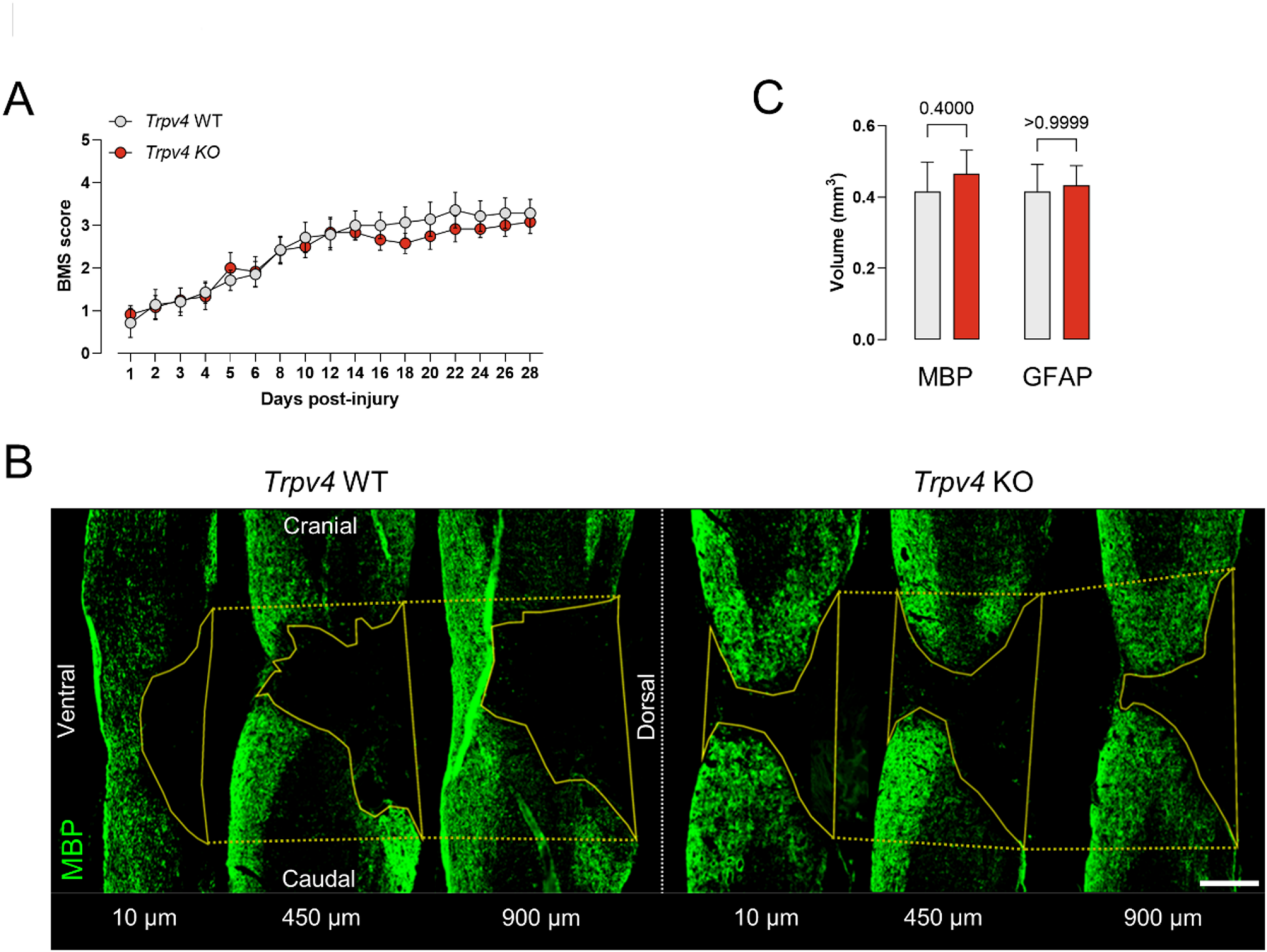
Full absence of TRPV4 does not contribute to recovery after spinal cord injury. (**A**) Functional recovery of 12-week-old *Trpv4* WT (*n* = 6) and *Trpv4* KO (*n* = 6) mice over 28 days after contusion injury at T8, of one individual experiment. (**B**) Overview of MBP around the lesion of a sagittal spinal cord section at 10, 450, and 900 μm depth. (**C**) Quantification of lesion volume surrounded by MBP and GFAP^+^ astrocytes at 28 dpi. Two-way ANOVA (**A**), Mann–Whitney* U* test (**C**). Data are presented as means ± SEM. Scale bar: 1 mm. WT, wild-type; KO, knockout; T8, thoracic level 8; MBP, myelin basic protein; GFAP, glial fibrillary acidic protein; dpi, days post-injury; BMS, Basso Mouse Scale.

Finally, a contusion SCI surgery was performed in 12-week-old *Trpv4* WT and *Trpv4* KO mice to exclude that the heterozygosity of *Cx3cr1* was responsible for the unexpected absence of effect. Animal follow-up was assessed until 28 dpi to evaluate locomotor recovery, and both groups reached a BMS score of 3 (plantar placing of the paw with or without weight support) at 28 dpi (Fig. 5A). No significant differences were found in functional recovery between *Trpv4* WT and *Trpv4* KO mice. At the histological level, we quantified the spinal lesion volume using MBP and GFAP. Over a whole sagittal-sliced spinal cord, specific slices were selected to cover the whole lesion with a range of 900 μm (Fig. 5B). The volume of each spinal cord lesion was calculated by measuring the area of each slice and thickness across all slices, as described previously^39^. No differences were observed in the lesion volume of both MBP and GFAP, respectively (Fig. 5C).

## Discussion

After SCI, microglia can play either a beneficial or detrimental role, depending on the balance between their pro- and anti-inflammatory functions^3,5,6,8,9^. Modulating microglial activity towards an optimal level may contribute to improved injury repair. We and others have recently identified TRPV4 as a key regulator of microglial migration, motility, and morphology through its interaction with the actin and tubulin cytoskeleton^17,19,21^. Furthermore, TRPV4 deficiency has been associated with a protective effect in SCI repair, leading to reduced microgliosis^22^. Therefore, targeting TRPV4 to fine-tune microglial activity may potentially improve SCI outcomes.

To investigate the cell-specific contribution of TRPV4 in SCI recovery, we developed two phagocyte-specific *Trpv4* KO mouse models and examined their impact on injury repair after SCI. These models included a tamoxifen-inducible cKO and bone marrow chimera, the latter combining BMT with PLX5622 treatment. This approach facilitated the repopulation of the spinal cord with transplanted myeloid cells, which acquired a microglia-like phenotype^37,40^. Previous studies indicate a high turnover of resident microglia in the spinal cord^37, ^a finding that we have corroborated in both *Trpv4* WT and *Trpv4* KO bone marrow cells (Fig. 2C). However, we recognize that peripherally derived macrophages may infiltrate the spinal cord while maintaining distinct phenotypic identities^36^. Given that both microglia and macrophages express TRPV4^35,41^, we have designated our models as phagocyte-specific *Trpv4* KO models.

Despite these targeted interventions, both the tamoxifen-induced *Trpv4* cKO model (Fig. 1B) and the phagocyte-specific bone marrow chimera (Fig. 3A) exhibited locomotor recovery comparable to control groups at acute and intermediate time points post-injury. Similarly, no significant differences were observed in microgliosis acutely after injury (Fig. 3C). Locomotor recovery, lesion size, scar formation, microgliosis, and astrogliosis were assessed in full TRPV4-depleted animals with (Fig. 4) and without (Fig. 5) heterozygous eGFP expression under the Cx3cr1 promoter at 28 dpi without significant differences between groups. To note, heterozygous models of *Cx3cr1* have been used in SCI studies and in several other studies to examine microglia physiology, demonstrating no confounding effect by the heterozygous expression of *Cx3cr1*^4,5,42–45^.

While our study found no evidence of a protective effect from either phagocyte-specific or systemic TRPV4 deficiency in SCI recovery (Fig. S2), previous research has suggested otherwise^22^. However, comparing these findings is challenging due to methodological differences. For instance, unlike Kumar et al., who sourced *Trpv4* WT and *Trpv4* KO mice from different suppliers, we exclusively used littermates to minimize genetic variability^46^. Additionally, the induction of SCI was performed using different techniques: Kumar et al. employed a weight-drop compression model (20 g/min at the thoracic level 10 (T10)), whereas we utilized a contusion model (85 kdyn at T8). Although both models replicate key aspects of human SCI pathophysiology, differences in impact dynamics and anatomical location may contribute to the contrasting results^47–49^.

Similar inconsistencies have been observed in TRPV4 research related to multiple sclerosis, where both beneficial and detrimental effects of TRPV4 inhibition on inflammation and myelination have been reported^50,51^. This suggests that subtle variations in immune context could influence functional recovery in experimental models. While TRPV4 activity is often associated with a pro-inflammatory response, it has also been implicated in immune regulation^35^. For instance, in lung macrophages, TRPV4 is required for phagocytosis and can modulate pro-inflammatory cytokine secretion in response to extracellular matrix stiffness^52,53^. This dual role could explain discrepancies across studies. An additional source of variability in studies concerning TRPV4’s role in a SCI response might be the use of genetic versus pharmacological models.

In conclusion, our findings indicate that TRPV4 in both bone marrow–derived and CNS-resident myeloid cells does not significantly influence functional recovery following SCI. The lack of improvement in both phagocyte-specific and systemic *Trpv4* KO models suggests that, despite its role in microglial functions, TRPV4 activity in SCI recovery is either non-essential or compensated by alternative molecular pathways. Further research is needed to determine whether selective TRPV4 modulation could yield therapeutic benefits in specific contexts.

## Materials and methods

### Animals

The *Trpv4* WT, *Trpv4* KO, *Trpv4* WT *Cx3cr1*^*eGFP/+*^, and *Trpv4* KO *Cx3cr1*^*eGFP/+*^ mice used in this study were obtained by in-house breeding. *Trpv4* KO mice were obtained thanks to the Laboratory of Ion Channel Research at KU Leuven (generated as described previously)^54^. *Cx3cr1*^*eGFP/eGFP*^ mice were provided by the European Mouse Mutant Archive (EMMA) Institute with the approval of Steffen Jung (Weizmann Institute of Science)^55^. These mice express eGFP under the Cx3cr1 promoter. The tamoxifen-inducible cKO model was obtained by breeding *Trpv4* floxed mice (*Trpv4*^*lox/lox*^), generated as described previously^56^, with *Cx3cr1*^*CreER/CreER*^ mice (EMMA Institute)^57^. Female mice were used for all experiments and were housed on a 12 h light/dark cycle in a standard animal care facility, with access to food and water *ad libitum*. Housing and experiments were conducted following the guidelines of the Belgian Law and the European Council Directive and with the approval of the Ethical Committee on Animal Research of Hasselt University (ID: 202160).

#### Ethics declaration

All experimental protocols were approved by the Ethical Committee on Animal Research of Hasselt University. All methods were carried out in accordance with relevant guidelines and regulations. All methods are reported in accordance with ARRIVE guidelines.

### Phagocyte-specific *Trpv4* KO mouse models

#### Tamoxifen-inducible cKO model

Female *Trpv4*^*lox/lox*^ mice^56^ (10-week-old) with heterozygous *Cx3cr1-CreER* expression received two intraperitoneal (i.p.) tamoxifen injections (57 mg/kg body weight, Sigma-Aldrich, Belgium, dissolved in corn oil) with 2 days apart (Fig. 1A). In addition, two control groups were included: (1) *Trpv4*^*lox/lox*^ *Cx3cr1*^*CreER/+*^ animals receiving corn oil injections; (2) *Trpv4*^*lox/lox*^ without *Cx3cr1-CreER* receiving tamoxifen injections to rule out possible toxicity of the compound^58^. Contusion-induced SCI was performed two weeks after injections (Fig. 1A).

Validation of the cKO model was performed using PCR on genomic DNA extracted from peritoneal macrophages and CD11b^+^ primary microglia isolated from *Trpv4*^*lox/lox*^ *Cx3cr1*^*CreER/+*^ animals two weeks after receiving corn oil or tamoxifen injections, as described above (Fig. S1). For the isolation of peritoneal macrophages, mice were sacrificed, and the peritoneal cavity was injected with phosphate-buffered saline (PBS) using a 27-gauge needle. The abdominal wall was gently agitated to dislodge peritoneal cells, which were then collected using a fresh 25-gauge needle^59^. CD11b^+^ primary microglia were isolated from dissected brains, as previously described^60^. Meninges were removed, and after homogenization, the resulting single-cell suspension was selected for CD11b^+^ cells via magnetic bead separation (Miltenyi Biotec, Germany). Genomic DNA was extracted from the cells using the KAPA Mouse Genotyping kit (KAPA Biosystems KK7352, Roche, Switzerland), according to the manufacturer’s instructions. Gene-specific primers were designed in exons 5 and 7 of the *Trpv4* gene to encompass the excision region. The primers used for genotyping were as follows: Forward (5’-GCTCTGGAGAAAGTTCACAC-3’) and Reverse (5’-CATAGTCTGGCTCCTAACGA-3’). Expected amplicon sizes were 650 bp for tamoxifen-induced recombination of *Trpv4* and 1276 bp for the corn oil-injected control (Fig. S1). Although protein level validation of TRPV4 was not performed, TRP channels are known to have a rapid turnover rate^61,^ suggesting that TRPV4 should not be expressed on the membrane after 14 days.

##### Bone marrow chimeras

Bone marrow chimeras were generated as described by Cronk et al.^36^. Briefly, 10-week-old female *Trpv4* WT and *Trpv4* KO mice underwent myeloablative therapy by sub-lethal total body γ-radiation (8 Gy) (Fig. 2A). Half of these animals received bone marrow transplants from *Trpv4* WT *Cx3cr1*^*eGFP/+*^ donors, while the others received bone marrow transplants from *Trpv4* KO *Cx3cr1*^*eGFP/+*^ donors. Donor cells (5 × 10^6^ cells/mouse) were freshly isolated from 10-week-old mice and inoculated into the host through the tail vein as described in^62^. After the BMT, mice were kept in sterile cages and treated for two weeks with antibiotics (Neomycin Sulfate (100 mg/ml, Gibco, USA) and Polymyxin B sulfate (60.000 units/ml, Sigma-Aldrich), provided *ad libitum* in drinking water). One week after transplantation, mice were fed with chow supplemented with colony-stimulating factor 1 receptor inhibitor PLX5622 (1.2 g/kg chow, Chemgood, USA) for two weeks. Contusion-induced SCI was performed 13 weeks post-transplantation (Fig. 2A).

### Contusion-induced SCI

At the age of 12 weeks (cKO) or 23 weeks (bone marrow chimeras), mice received a contusion-induced SCI. Mice were sedated with 3% isoflurane (IsoFlo, Abbot Animal Health, Belgium), maintained at 1.5%, and subjected to a partial laminectomy at T8 to expose the spinal cord. The vertebral column was stabilized and supported by Allis clamps, and a severe contusion (85 kdyn) was made using the Infinite Horizon Impactor (Precision Systems and Instrumentation Impactors, USA). Muscles were sutured, and the skin was closed with wound clips (BD Medical, Belgium). Post-operative recovery included blood-loss compensation by i.p. injection of 1 ml glucose (20%) and subcutaneous post-operative pain management (Buprenorphine; 0.1 mg/kg body weight, Temgesic) until 3 dpi. Animals were allowed to recover in a temperature-controlled chamber (33 °C) until awake. Bladders were emptied manually daily until the mice ceased to retain urine. Humane endpoints were defined as: > 25% weight loss compared to highest measured bodyweight (weakly measured), dehydration (observational), a rapidly-spreading deadly infection (quarantined or euthanized depending on the illness), chronic pain (more than 48 h, observation of piloerection, prolapse, and a hunched back), urinary system infection (dark green/red urine > two days and untreatable with Baytril), bladder rupture (due to daily voiding by researcher), or self-mutilation (loss of limb or tail). In case a humane endpoint is reached, the mice will be sacrificed by means of cervical dislocation.

### Functional recovery

The hindlimb locomotion recovery was assessed using the Basso Mouse Scale (BMS) score^46^. Mice were scored by an investigator blinded to the randomized experimental groups using a 10-point locomotor rating scale (9 = normal locomotion; 0 = complete hind limb paralysis). During the first week after injury, mice were scored daily. From the start of the second week until the end of the observation period (28 dpi), mice were examined every other day. Mice with a BMS score of > 2 at 3 dpi or a BMS score of 0 at day 28 were excluded from the study.

### Immunofluorescence and imaging

At different experimental time points (3, 7, and 28 dpi), animals were euthanized with an overdose of i.p. dolethal (200 mg/kg, Vetiquinol B.V., Netherlands) and were transcardially perfused with Ringer solution, followed by 4% paraformaldehyde (PFA) for tissue fixation. The spinal cords were isolated and post-fixed overnight in 4% PFA-5% sucrose at 4 °C. After incubation in 30% sucrose in PBS for 72 h, the spinal cords were embedded in Tissue-Tek O.C. and cryopreserved by freezing them with the isopentane-liquid nitrogen method. Serial sagittal sections (six sections per microscope slide, minimum five slides per animal) 10 μm thick were prepared using a cryostat (Leica CM3050, Leica Biosystems, Belgium), totaling 900 μm of tissue. Consecutive samples were blocked with 10% protein block (Dako, Agilent, USA) in PBS and stained overnight at 4 °C with primary antibodies to visualize the glial scar (GFAP; Mouse Anti-GFAP, 1:200, Sigma-Aldrich), extracellular components of the scar (Laminin; Rabbit Anti-Laminin, 1:500, Abcam, UK) and the demyelinated area (MBP; Rat Anti-MBP, 1:250, Merck Millipore, Belgium). Microglia were visualized via the endogenous expression of eGFP under the Cx3cr1 promoter. Secondary antibodies were Alexa Fluor 568 goat anti-mouse (1:250, Invitrogen, Belgium), Alexa 488 goat anti-rat (1:250, Invitrogen), and Alexa Fluor 555 goat anti-rabbit (1:500, Invitrogen), and were incubated for 1 h at room temperature. Nuclei were counterstained with DAPI (Sigma-Aldrich) for 15 min. Images were acquired using a widefield microscope (Nikon Eclipse Ti2-E) with a CFI Plan-ApoChromat Lambda 20x/0.75 objective and analyzed using Fiji software^63^.

Volumetric analysis of GFAP and MBP was performed by selecting specific slices to cover the whole lesion with a range of 900 μm. The volume of each spinal cord lesion was calculated by measuring the area of each slice and thickness across all slices, as described previously^39^.

### Statistical analyses

GraphPad Prism 9 (GraphPad Software, USA) was used for statistical analysis. Normal distribution and equal variances of the residuals were assessed with the Shapiro-Wilk test and Brown Forsythe, respectively. Hindlimb locomotion recovery was analyzed using a two-way ANOVA for repeated measurements with a Bonferroni *post hoc* test for multiple comparisons. Lesion hallmarks were analyzed using an Unpaired* T*-test, a Multiple unpaired* T*-test, or a Multiple Mann–Whitney* U* test. Data were represented as mean ± SEM. * P*-values ≤ 0.05 were considered significant. The respective statistical analysis used to analyze the data is mentioned in the figure legends.

## Electronic supplementary material

Below is the link to the electronic supplementary material.

Supplementary Information 1

## Data Availability

Data will be available upon reasonable request to the corresponding authors.
