# Supplementary material for: Loss of TRPV4 is insufficient to promote repair in a spinal cord injury contusion model
Source: Sci Rep. 2025 Jul 23;15:26757. doi: 10.1038/s41598-025-12372-1 (PMC12287530; doi:10.1038/s41598-025-12372-1)
Supplement: Supplementary file 1 — Supplementary Information 1 [file 41598_2025_12372_MOESM1_ESM.pdf]

Supplementary information

Loss of TRPV4 is insufficient to promote repair in a spinal cord injury contusion model

Melanie Mertens, Sofie Kessels, Naomi Veeningen, Elle E.M. Scheijen, Femke Mussen, Amber Delbroek, Jana Van Broeckhoven, Yeranddy A. Alpizar, Bert Brône

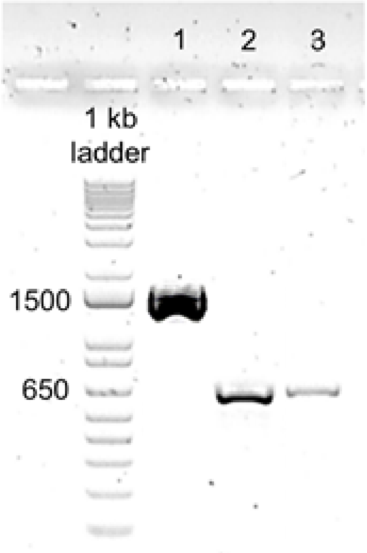

Fig. S1: Validation of the tamoxifen-inducible *Trpv4* conditional knockout model. PCR results of genomic DNA extracted from peritoneal macrophages isolated from *Trpv4*$^{lox/lox}$ *Cx3cr1*$^{CreER/+}$ animals two weeks after receiving corn oil (Lane 1) or tamoxifen (Lane 2) injections. Lane 3 corresponds to CD11b^{+} primary microglia isolated from *Trpv4*$^{lox/lox}$ *Cx3cr1*$^{CreER/+}$ animals after receiving tamoxifen injections. The shift from 1276 bp to 650 bp indicates efficient tamoxifen-induced recombination of *Trpv4*.

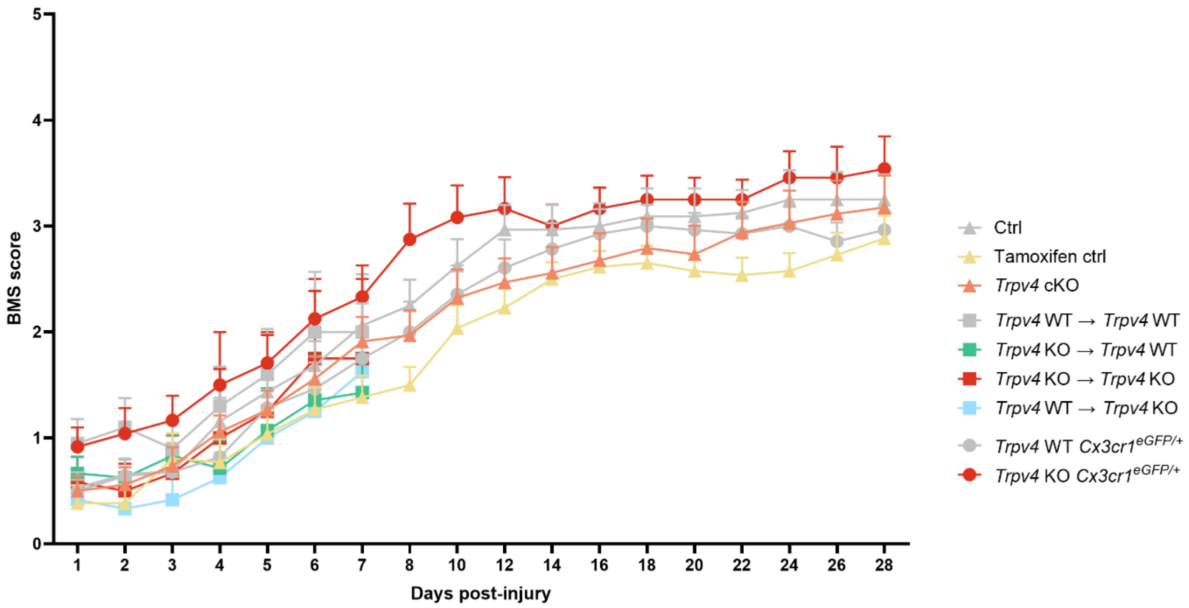

Fig. S2: Overview of the functional recovery of three animal models after contusion injury 1. Tamoxifen inducible model: (▲) *Trpv4*$^{lox/lox}$ *Cx3cr1*$^{CreER/+}$ animals receiving corn oil injections (Ctrl); (▲) *Trpv4*$^{lox/lox}$ without *Cx3cr1-CreER* receiving tamoxifen injections (Tamoxifen ctrl); (▲) *Trpv4*$^{lox/lox}$ *Cx3cr1*$^{CreER/+}$ animals receiving tamoxifen injections (57 mg/kg body weight) (*Trpv4* cKO). **2. BMT model:** (■) full WT (*Trpv4* WT → *Trpv4* WT) n=5; (■) phagocyte-specific *Trpv4* KO (*Trpv4* KO *Cx3cr1*$^{eGFP/+}$ → *Trpv4* WT) n=7; (■) full *Trpv4* KO (*Trpv4* KO *Cx3cr1*$^{eGFP/+}$ → *Trpv4* KO) n=2; (■) TRPV4-expressing phagocytes in a TRPV4-deficient host (*Trpv4* WT *Cx3cr1*$^{eGFP/+}$ → *Trpv4* KO) n=4. **3. Constitutive *Trpv4* KO (●)***Trpv4* WT *Cx3cr1*$^{eGFP/+}$ (n=14) and (●) *Trpv4* KO *Cx3cr1*$^{eGFP/+}$ (n=12) mice. Data are presented as means + SEM. Ctrl; control, cKO; conditional knockout, BMT; bone marrow transplantation, WT; wild type, KO; knockout, eGFP; enhanced green fluorescent protein, BMS; Basso Mouse Scale.
